# Comparative study on incorporation of three recombinant human α-galactosidase A drugs (agalsidases) into cultured fibroblasts and organs/tissues of Fabry mice

**DOI:** 10.1016/j.ymgmr.2024.101118

**Published:** 2024-07-15

**Authors:** Takahiro Tsukimura, Tomoko Shiga, Tadayasu Togawa, Hitoshi Sakuraba

**Affiliations:** a Department of Functional Bioanalysis, Meiji Pharmaceutical University, 2-522-1 Noshio, Kiyose, Tokyo, 204-8588, Japan; b Department of Clinical Genetics, Meiji Pharmaceutical University, 2-522-1 Noshio, Kiyose, Tokyo, 204-8588, Japan

**Keywords:** Fabry disease, α-Galactosidase A, Enzyme replacement therapy, Cation-independent mannose 6-phosphate receptor, Globotriaosylceramide, Globotriaosylsphingosine

## Abstract

Enzyme replacement therapy (ERT) with recombinant human α-galactosidase A (α-Gal A) drugs (agalsidases) has been successfully used for treatment of Fabry disease, and three kinds of agalsidases are now available in Japan. To compare the biochemical characteristics of these drugs, especially focusing on their incorporation into cultured fibroblasts and organs/tissues of Fabry mice, we performed in vitro, cell, and animal experiments.

The results revealed that there were no differences in the kinetic parameters and enzyme activity between these agalsidases. But their affinity for domain 9 of cation-independent mannose 6-phosphate receptor (CI-M6PR), which exists in various cells, was higher in the order: agalsidase beta biosimilar 1 (agalsidase beta BS) > agalsidase beta > agalsidase alfa, which almost coincided with the experimental results regarding the efficiency of their incorporation into cultured fibroblasts derived from a Fabry mouse. The results of animal experiments using Fabry mice revealed that the incorporation of the agalsidases into the kidneys and heart, where CI-M6PRs are widely distributed, was efficient in the order: agalsidase beta/agalsidase beta BS > agalsidase alfa, which reflected the degree of reduction of glycosphingolipids accumulated in the organs/tissues. On the other hand, no differences in the efficiency of their uptake or reduction of the accumulated substances were observed in the liver, probably due to asialoglycoprotein receptors expressed on the surface of hepatocytes.

This information will be useful for making a suitable ERT plan for individual Fabry patients with various backgrounds and for developing new ERT drugs in the future.

## Highlights

• Characteristics of agalsidases (α, β, and βBS) authorized in Japan were compared.
• The affinity of agalsidases for domain 9 of CI-M6PR was higher in the order: βBS > β > α.
• The efficiency of uptake by cultured fibroblasts was also higher in the order: βBS > β > α.
• The uptake of β/βBS by the kidneys and hearts of mice was higher than that of α.
• The Gb3 cleavage in the kidneys was more efficient on ERT with β/βBS than that of α.

## 1 Introduction

Fabry disease (OMIM 301500) is an X-linked genetic disease caused by deficient α-galactosidase A (α-Gal A) activity [1]. The enzyme defect, based on pathogenic variants in the *GLA* gene, results in storage of glycosphingolipids, predominantly globotriaosylceramide (Gb3) and its derivative, globotriaosylsphingosine (Lyso-Gb3), in various cell types, leading to multi-system disease. This causes a progressive disorder involving such as renal failure, cardiac myopathy and rhythm disturbances, and stroke, being potentially life-threatening.

Since 2001, enzyme replacement therapy (ERT) with recombinant human α-Gal A drugs (agalsidases) has become available as the standard therapy for this disease, and is widely used. Regarding the effect of ERT, many reports have described that it stabilizes, delays or prevents progressive organ damage and ameliorates the symptoms [[1], [2], [3], [4]], although the response to the drugs differs depending on the timing of initiation of ERT, clinical phenotype of a patient, and stage of disease [5].

In Japan, three kinds of recombinant human α-Gal A drugs (agalsidase alfa, agalsidase beta, and agalsidase beta biosimilar 1 (agalsidase beta BS)) have received marketing authorization. Agalsidase alfa [6] produced in human fibroblasts is given intravenously every 2 weeks at a dose of 0.2 mg/kg body weight, and agalsidase beta [7] and agalsidase beta BS [8] produced in Chinese hamster ovary cells, fortnightly at a dose of 1.0 mg/kg body weight. They are incorporated into cells from blood by endocytosis through various receptors including cation-independent mannose 6-phosphate receptors (CI-M6PRs) [9,10], asialoglycoprotein receptors [11,12], mannose receptors [13], megalin [14,15], and sortilin [16,17], exhibiting different distributions depending on the cell type. Among them, CI-M6PR exists on the cell membranes of various cells and plays an important role in the uptake of recombinant human α-Gal A drugs [9,10].

Comparative studies targeting these agalsidases have been performed with a combination of agalsidase alfa vs. agalsidase beta [18,19], and agalsidase beta vs. agalsidase beta BS [20]. However, there is no report describing the results of biochemical characterization of these three agalsidases performed under the same experimental conditions. To select an appropriate treatment for individual Fabry patients with various backgrounds, it is important to obtain information about the biochemical differences between the drugs.

In this study, we tried to characterize the agalsidases by in vitro, cellular, and animal experiments under the same experimental conditions, especially focusing on their cellular uptake through CI-M6PRs, and evaluated the efficacy of ERT with these agalsidases.

## 2 Materials and methods

### 2.1 Recombinant human α-Gal A drugs (agalsidases)

Three kinds of recombinant α-Gal A drugs: agalsidase alfa (Replagal®, Takeda Shire, Cambridge, MA), agalsidase beta (Fabrazyme®, Sanofi Genzyme, Cambridge, MA), and agalsidase beta BS (Agalsidase beta BS®, JCR Pharmaceuticals Co. Ltd., Ashiya, Japan) were used for the valid comparison in this study.

### 2.2 Cultured cells

Cultured fibroblasts derived from skin tissue of a Fabry mouse (F666) [21] were used for cell experiments in this study.

### 2.3 Animals

Fabry mice (*Gla* knock-out mice, C57BL/6 x 129Svj hybrid background) denoted by Drs. A.B. Kulkarni and T. Oshima (National Institute of Dental Research, National Institute of Health) [22,23] were bred and maintained at the Animal Center of our university. The Fabry mice and wild-type C57BL/6 mice, as a control, were used for animal experiments in this study. The experiments were approved by the animal ethics committee of our university.

### 2.4 Measurement of enzyme activity

Enzyme activity of α-Gal A in the samples (agalsidases, cultured fibroblasts, and organs/tissues of mice) was fluorometrically measured using 4-methylumbelliferyl-α-D-galactopyranoside (Calbiochem, La Jolla, CA) as a substrate, and *N*-acetyl-D-galactosamine (GalNAc; Sigma-Aldrich, St. Louis, MO) as an inhibitor of α-*N*-acetylgalactosaminidase (α-galactosidase B), according to the previous report [24]. The fluorescence of 4-methylumblliferone released on the enzyme reaction was measured with a Varioskan LUX (Thermo Scientific, Rockford, IL) at excitation and emission wavelengths of 365 nm and 450 nm, respectively. The enzyme activity is expressed as nmol/h/mg protein, and the protein determination was performed with a Micro BCA Protein Assay kit (Thermo Scientific), using bovine serum albumin (BSA) as the standard.

### 2.5 Determination of enzyme kinetic parameters

In the enzyme kinetic study, α-Gal A assaying was performed three times at each substrate concentration (1, 2, 4, 6, and 8 mmol/L enzyme reaction mixture), and the mean values were calculated for each agalsidase. Then, the maximum velocity (*Vmax*) and the Michaelis constant (*Km*) of each agalsidase were determined based on Lineweaver-Burk plotting using the calculated data. For the enzyme assaying in the kinetic study, GalNAc, as the inhibitor of α-*N*-acetylgalactosaminidase, was excluded from the enzyme reaction mixture, and BSA (200 μg/mL) was added as a stabilizer of the enzyme protein.

### 2.6 Determination of binding-kinetics parameters on complex formation between agalsidases and domain 9 of CI-M6PR

Affinity of the agalsidases with recombinant human domain 9 of CI-M6PR [25], donated by Dr. Y. Chiba (Glycoscience and Glycotechnology Research Group, Biotechnology Research Institute for Drug Discovery, National Institute of Advanced Industrial Science and Technology), was examined by the surface plasmon resonance (SPR) method using a Biacore X100 system (Cytiva, Tokyo, Japan). Domain 9 of CI-M6PR (ligand) was coupled to a certified grade CM5 chip (Cytiva) using the amine coupling kit supplied by the manufacturer, and then the unreacted species on the surface of the chip were blocked with ethanolamine. Then, determination of the binding rate constant (*k*_a_), dissociation rate constant (*k*_d_), and dissociation constant (*K*_D_) values for the agalsidases (analytes) was performed according to the manufacturer's methods. For the formation of the complex, the solution of agalsidases (agalsidase alfa: 2.5, 5, 10, 20, and 40 μg/mL; agalsidase beta: 0.5, 1, 2, 4, and 8 μg/mL; and agalsidase beta BS: 0.25, 0.5, 1, 2, and 4 μg/mL) were passed over the chip at a flow rate of 30 μL/min. For dissociation of the complex, HBS-EP buffer, pH 7.4, was passed over the chip on which the ligand/analyte complex was coated. The formation and dissociation of the complex were performed at 25 °C.

### 2.7 Examination of uptake of agalsidases by cultured fibroblasts derived from a Fabry mouse

To examine the uptake of agalsidases by F666 cells, the cells were distributed into the wells of a commercially available 12-well culture plate at the concentration of 1.0 × 10^5^ cells/well, and then cultured in Ham's F-10 medium containing fetal bovine serum and antibiotics for 24 h in a humidified incubator flushed continuously with a 5% CO_2_–95% air mixture. Then the culture medium was changed to F-10 medium containing 0, 0.5, 1, and 3 μg/mL of each agalsidase, and the cells were further cultured for 24 h. After the culture, the cells were harvested, washed three time with phosphate-buffered saline (PBS), pH 7.4, and then collected as a pellet by centrifugation. An appropriate amount of 20 mmol/L MES buffer, pH 6.0, containing protease inhibitors was then added to the pellet, and the cells were disrupted with ultrasonic waves. Then, the cell-associated α-Gal A activity was measured using the cell homogenates as samples. Furthermore, to examine the involvement of CI-M6PR in endocytosis, mannose 6-phosphate (M6P) was added at the concentration of 5 mmol/L to the medium containing 3 μg/mL of each agalsidase, and then the cells were cultured for 24 h and the intracellular α-Gal A activity was measured under the conditions described above.

### 2.8 Examination of the effects of administered agalsidases on increases of enzyme activity and cleavage of accumulated glycosphingolipids in organs/tissues of Fabry mice

Fabry mice (10-week-old males) were used for the animal experiments. One mg/kg body weight of agalsidase alfa, agalsidase beta, and agalsidase beta BS were independently injected into the Fabry mice via a tail vein. The mice were sacrificed 24 h after administration of the drugs. The livers, kidneys, and hearts were removed after perfusion with PBS. Then, the homogenates of the organs/tissues were used as samples for measurement of α-Gal A activity and determination of the content of Gb3/Lyso-Gb3. As controls, Fabry mice and wild-type ones without treatment were used.

The enzyme activity in the organs/tissues was measured by the fluorometric assay method described above.

The contents of Gb3 and Lyso-Gb3 in the organs/tissues were determined by means of liquid chromatography (LC)-tandem mass spectrometry (MS/MS) using Gb3 (C17:0) (Matreya L.L.C., Pleasant Gap, PA) and stable isotope-labelled Lyso-Gb3 (Nard Institute, Kobe, Japan) as internal standards, according to the method described previously [26]. Briefly, glycosphingolipids in the tissue homogenates were extracted with chloroform/methanol (vol., 1:2), and then separated by LC using a Unison UK-C8 column (20 × 3 mm, I.D., 3 μm; Imtakt Co., Kyoto, Japan). Then, Gb3 isoforms and Lyso-Gb3 in the samples were detected and quantified by MS/MS with a LCMS-8040 triple quadrupole mass spectrometer (Shimadzu, Kyoto, Japan) equipped with an electrospray ionization interface in the positive-ion mode. The total Gb3 content in the samples was calculated from the sum of the Gb3 isoforms.

### 2.9 Statistical analysis

Date are essentially expressed as means *±* standard deviation (SD). Statistical analysis was performed using Student's *t*-test. Values were considered statistically significant at *P* < 0.05.

## 3 Results

### 3.1 Enzyme activity and kinetic parameters

Enzyme activity and kinetic parameters of the agalsidases were determined by fluorometric enzyme assaying and Lineweaver-Burk plotting, respectively. The results are shown in Table 1. No significant differences in the values for *Vmax*, *Km,* and enzyme activity were observed between agalsidase alfa, agalsidase beta, and agalsidase beta BS.

**Table 1:** Enzyme kinetics of agalsidases.

	Enzyme activity (mmol/h/mg protein)	*Vmax* (mmol/h/mg protein)	*Km* (mmol/L)
Agalsidase alfa	3.2 ± 0.1	6.6	4.0
Agalsidase beta	3.4 ± 0.1	5.9	3.9
Agalsidase beta BS	3.4 ± 0.1	6.3	4.4

The enzyme assaying and protein determination were performed using the agalsidases as samples.

The enzyme activities are expressed as means ± standard deviation (*n* = 3).

The values for *Vmax* and *Km* of each agalsidase were determined based on Lineweaver-Burk plotting.

*Vmax:* maximum velocity; *Km:* Michaelis constant.

### 3.2 Kinetics of complex formation between agalsidases and domain 9 of CI-M6PR

The affinity of agalsidases with domain 9 of CI-M6PR was biophysically examined by means of SPR biosensor assays. The results are shown in Table 2. Based on the values for *k*_a_ and *k*_d_ obtained, the *K*_D_ value was determined for each agalsidase, and the results revealed that the affinity with domain 9 of CI-M6PR is higher in the order: agalsidase beta BS > agalsidase beta > agalsidase alfa. The data suggested that the stronger binding of agalsidase beta BS with domain 9 of CI-M6PR was due to both fast complex formation and late dissociation.

**Table 2:** Binding affinity of agalsidases for domain 9 of cation-independent mannose 6-phosphate receptor.

	*k*_*a*_ (L/mol·s × 10^5^)	*k*_*d*_ (1/s × 10^−3^)	*K*_D_ (mol/L × 10^−8^)
Agalsidase alfa	2.31 ± 0.18	13.9 ± 0.4	6.04 ± 0.42
Agalsidase beta	5.35 ± 0.11	9.72 ± 0.66	1.82 ± 0.14
Agalsidase beta BS	11.8 ± 0.8	7.87 ± 0.33	0.669 ± 0.042

The values for *k*_*a*_*, k*_d_*,* and *K*_D_ of each agalsidase are expressed as means ± standard deviation (n = 4).

*P* values for *k*_*a*_, *k*_*d*_, and *K*_*D*_ < 0.05 (agalsidase alfa vs. agalsidase beta; agalsidase beta vs. agalsidase beta BS; and agalsidase alfa vs. agalsidase beta BS).

*k*_*a*_*:* rate constant of complex formation.

*k*_*d*_: rate constant of complex dissorciation.

*K*_*D*_: equilibrium constant.

### 3.3 Uptake of agalsidases by cultured fibroblasts derived from a Fabry mouse

**Fig. 1:**
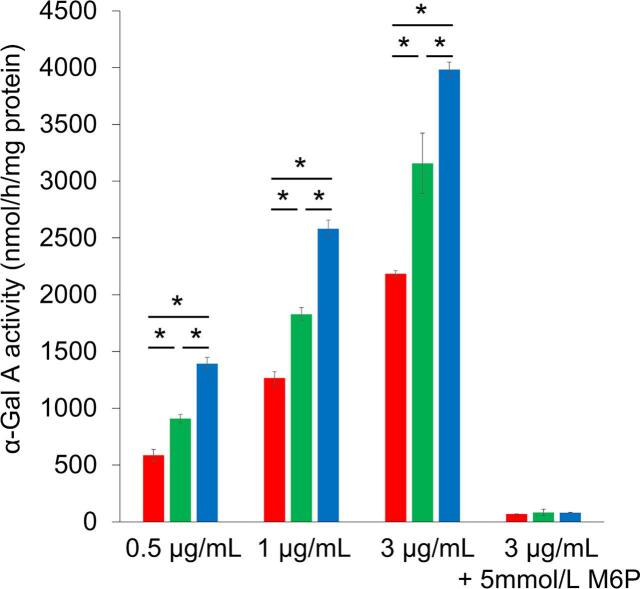
Uptake of agalsidases by cultured fibroblasts derived from a Fabry mouse (F666). F666 cells were cultured for 24 h with medium containing 0.5, 1, and 3 μg/mL of each agalsidase with or without 5 mmol/L of mannose 6-phosphate, and then cell-associated α-galactosidase A activity was fluorometrically measured and protein determination was performed using cell homogenates of the cultured fibroblasts as samples. The red, green, and blue columns exhibit the cell-associated enzyme activities in the cases of administration of agalsidase alfa, agalsidase beta, and agalsidase beta BS, respectively (vertical axis). Error bars represent means ± standard deviation (*n* = 4). The enzyme activity in non-treated F666 cells (background) was almost nil (1 nmol/h/mg protein), although that in non-treated fibroblasts from a wild-type mouse (F665) was 93 nmol/h/mg protein [21]. The horizontal axis shows the concentration of agalsidases in the culture medium. * *P* < 0.05.

The uptake of agalsidases by F666 cells was examined, and the results are summarized in Fig. 1. The α-Gal A activity in non-treated F666 cells was almost nil, and an apparent increase in cell-associated enzyme activity was observed after the culture in medium containing the agalsidases. The results revealed that these agalsidases were efficiently incorporated into F666 cells in the following order: agalsidase beta BS > agalsidase beta > agalsidase alfa, and that their incorporation into the cells was dose-dependent. When the cells were cultured in the medium containing both agalsidases and M6P, the increase in cell-associated enzyme activity almost completely disappeared, suggesting that the incorporation of agalsidases into F666 cells strongly depends on CI-M6PRs on the cell membrane.

### 3.4 Therapeutic effects of agalsidases on Fabry mice

**Fig. 2:**
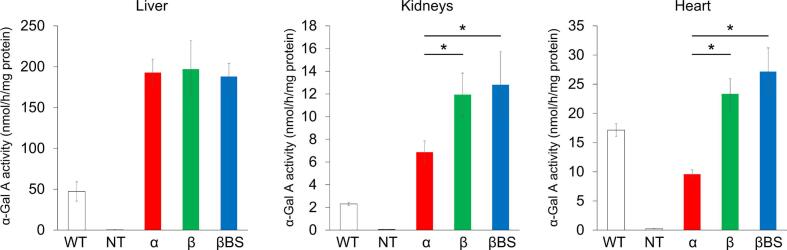
Effects of administration of agalsidases on the increase of α-galactosidase A (α-Gal A) activity in the organs/tissues of Fabry mice. One mg/kg body weight of agalsidases was independently administered to Fabry mice, and then the α-Gal A activity in the liver, kidneys, and heart was fluorometrically measured and protein determination was performed using the tissue homogenates as samples after 24 h. The white, gray, red, green, and blue columns represent the enzyme activities in the cases of non-treated wild-type mice (WT), non-treated Fabry ones (NT), and Fabry mice treated with agalsidase alfa (α), agalsidase beta (β), and agalsidase beta BS (βBS), respectively. Error bars represent means ± standard deviation (n = 4). * P < 0.05.

**Fig. 3:**
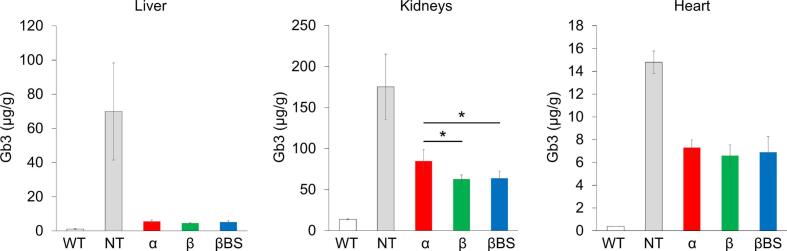
Effects of administration of agalsidases on the reduction of globotriaosylceramide (Gb3) accumulated in the organs/tissues of Fabry mice. One mg/kg body weight of agalsidases was independently administered to Fabry mice, and then the contents of Gb3 accumulated in the liver, kidneys, and heart were measured by liquid chromatography-mass spectrometry after 24 h. The white, gray, red, green, and blue columns represent the contents of Gb3 in the cases of non-treated wild-type mice (WT), non-treated Fabry mice (NT), and Fabry mice treated with agalsidase alfa (α), agalsidase beta (β), and agalsidase beta BS (βBS), respectively. Error bars represent means ± standard deviation (n = 4). * P < 0.05.

**Fig. 4:**
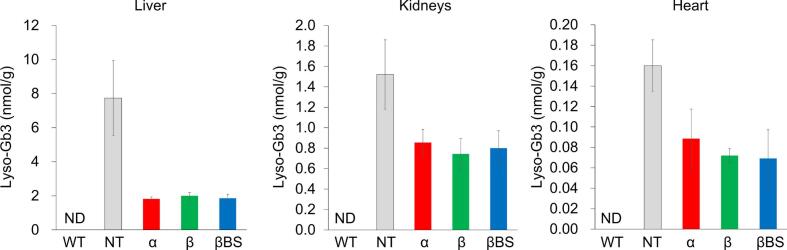
Effects of administration of agalsidases on the reduction of globotriaosylsphingosine (Lyso-Gb3). One mg/kg body weight of agalsidases was independently administered to Fabry mice, and then the contents of Lyso-Gb3 accumulated in the liver, kidneys, and heart were measured by liquid chromatography-mass spectrometry after 24 h. The white, gray, red, green, and blue columns represent the contents of Lyso-Gb3 in the cases of non-treated wild-type mice (WT), non-treated Fabry mice (NT), and Fabry mice treated with agalsidase (α), agalsidase beta (β), agalsidase beta BS (βBS), respectively. Error bars represent means ± standard deviation (n = 4). ND: not detected.

To examine the therapeutic effects of the agalsidases on Fabry mice, they were independently injected into tail veins of the mice. At 24 h after administration of a single dose of each enzyme, the mice were sacrificed, and the α-Gal A activity and contents of Gb3/Lyso-Gb3 in the livers, kidneys and hearts were measured. The results are summarized in Fig. 2, Fig. 3, Fig. 4.

An apparent increase in the enzyme activity was observed in these organs and tissues, predominantly in the liver, after the administration of the agalsidases, and there were no significant differences in the enzyme activity in the liver after the administration between the agalsidases. On the other hand, the increases in the enzyme activity in the kidneys and heart were significantly larger in the cases of administration of agalsidase beta/agalsidase beta BS than in that of agalsidase alfa. Although there was a tendency that the degree of increase in the enzyme activity was moderately larger in the case of administration of agalsidase beta BS than that of agalsidase beta, no statistical difference was observed between them (Fig. 2).

The accumulation of a large amount of Gb3 was observed in the organs and tissues, predominantly in the kidneys, of the untreated Fabry mice, compared with those of the wild type ones, and the administration of the agalsidases facilitated the degradation of Gb3 accumulated. Especially in the liver, the content of Gb3 decreased to <10% of the baseline on ERT, and no differences in the efficacy were observed between the agalsidases. In the kidneys, the contents of Gb3 accumulated decreased to 53, 37, and 37% of the baseline, on administration of agalsidase alfa, agalsidase beta, and agalsidase beta BS, respectively. A statistical difference in the efficacy of Gb3 degradation was observed between agalsidase beta/agalsidase beta BS and agalsidase alfa. In the heart, the contents of Gb3 accumulated decreased to 47, 39, and 41% of the baseline, on administration of agalsidase alfa, agalsidase beta, and agalsidase beta BS, respectively. The degree of reduction in Gb3 accumulated in the heart was moderately greater in the cases of administration of agalsidase beta/agalsidase beta BS than in that of agalsidase alfa, but a statistical difference was not observed between them (Fig. 3).

In these organs and tissues of the untreated Fabry mice, accumulation of Lyso-Gb3 was also observed in the following order: liver > kidneys > heart, although the contents of Lyso-Gb3 accumulated were significantly lower compared with those of Gb3. All the agalsidases administrated reduced the contents of Lyso-Gb3 accumulated in the organs and tissues. The content of Lyso-Gb3 in the liver decreased to 25–28% of the baseline after the administration of these agalsidases, and there were no differences in the efficacy between them. Those in the kidneys decreased to 61, 49, and 53% of the baseline, when agalsidase alfa, agalsidase beta, and agalsidase beta BS were injected, respectively. Those in the heart decreased to 56, 39, and 33% of the baseline after the injection of agalsidase alfa, agalsidase beta, and agalsidase beta BS, respectively. The degree of reduction in the contents of Lyso-Gb3 accumulated in the kidneys and heart on administration of agalsidase beta/agalsidase beta BS was moderately larger than in the case of agalsidase alfa, although no statistical difference was observed between them (Fig. 4).

## 4 Discussion

In this study, we compared biochemical characteristics of the agalsidases approved in Japan, especially those involved in cellular uptake and efficacy of ERT. Regarding these recombinant enzymes, the amino acid sequence, as well as the nucleotide sequence that encodes it, is identical to that of the natural form of human α-Gal A. However, the composition of the sugar chains posttranslationally attached to the enzyme molecules differs depending on the host cells which produce them and the culture condition.

Actually, there are no differences in the kinetic parameters and enzyme activity between the agalsidases, probably due to the identity of the protein moieties comprising them. On the other hand, as to the monosaccharide compositions of sugar chains of the agalsidases, many investigators have reported that there are differences between them [[18], [19], [20]]. According to these reports, the content of M6P residues, which are important for endocytosis via CI-M6PR, in the sugar chains is considered to be higher in the order: agalsidase beta BS > agalsidase beta > agalsidase alfa, as summarized in Supplementary Table 1.

The CI-M6PR is a huge molecule responsible for trafficking M6P-tagged lysosomal enzymes including α-Gal A. The extracellular region of the CI-M6PR has 15 homologous domains, including M6P-binding domains 3, 5, 9, and 15 [27]. It has been reported that not only the number of M6P residues but also the glycan structure influences the complex formation of M6P-tagged glycoproteins with CI-M6PR [[28], [29], [30]]. Thus, we biophysically examined the affinity of the agalsidases with domain 9 of CI-M6PR, which exhibits strong affinity with M6P residues, by means of SPR analysis. The agalsidases exhibited higher affinity for domain 9 of CI-M6PR in the following order: agalsidase beta BS > agalsidase beta > agalsidase alfa. The results of SPR analysis almost coincided with those of cell experiments. This suggests that the differences in the contents of M6P residues and glycan structures influenced the cellular uptake of the drugs. Actually, a glycoengineered lysosomal enzyme (avalglucosidase alfa) with improved cellular uptake has been developed to enhance the efficacy of ERT for Pompe disease [31,32].

Then, we examined the α-Gal A activity and contents of Gb3/Lyso-Gb3 in organs and tissues of Fabry mice after single dose administration of the agalsidases. The results revealed that large amounts of the enzymes injected were incorporated into the liver, and no significant differences in the reduction of Gb3/Lyso-Gb3 were observed between these agalsidases. As a lot of asialoglycoprotein receptors are expressed on the surface of hepatocytes of the liver [11,12], these receptors may be deeply involved in the uptake of large amounts of the drugs. On the other hand, a difference in the increase of enzyme activity in the kidneys and heart after administration of the drugs was observed between agalsidase beta/agalsidase beta BS and agalsidase alfa. As it has been reported that CI-M6PR is highly distributed in the kidneys and heart [33], the effective incorporation of agalsidase beta/agalsidase beta BS into these organs and tissues may be due to high affinity of these drugs for CI-M6PR. Cleavage of Gb3 in the kidneys was more efficient with the administration of agalsidase beta/agalsidase beta BS than with that of agalsidase alfa. Although there was a tendency that the cleavage of Gb3 in the heart and that of Lyso-Gb3 in the kidneys and heart were moderately more efficient with the administration of agalsidase beta/agalsidase beta BS compared with that of agalsidase alfa, no statistical differences were observed between them. Thus, the results of animal experiment were not necessarily associated with those of cell experiment using cultured fibroblasts, expressing a lot of CI-M6PRs on the cell surface. It suggests that the uptake mechanisms of the agalsidases in the organs and tissues cannot be simply evaluated by CI-M6PR system, because those consists of various cells having their own receptors for endocytosis as described in the “Introduction” part. Also the differences in biochemical properties and contents of the accumulated substances might have influenced the results.

As to the movement and function of the agalsidases incorporated into cells, the Drug Interview Forms of them [[34], [35], [36]] describe that “the drugs are transferred to lysosomes and degrade Gb3 accumulated, and then quickly hydrolyzed”. Regarding the stability of agalsidases in organs and tissues in Fabry mice, previous reports demonstrated that the time courses of enzyme activity in organs and tissues exhibited similar profiles between agalsidase alfa and agalsidase beta, although there are some differences in dosages and frequency of drug administration between them [37,38]. Furthermore, the Drug Interview Form of agalsidase beta BS describes that there are no significant differences in the distribution rate in organs and tissues of Fabry mice between agalsidase beta and agalsidase beta BS [36]. As to the stability of enzyme activity in plasma, we previously examined and revealed that there was no difference between agalsidase alfa and agalsidase beta [39].

This study has limitations. Our experiments were performed under certain limited conditions, and we report here the results of characterization concentrating on uptake of the drugs by cultured fibroblasts and organs/tissues of Fabry mice. We must consider the potential existence of species-specific mechanisms and take care when we try to apply the interpretation of the results of animal experiment to that of humans. Furthermore, we should recognize that each agalsidase has its own advantages and disadvantages for ERT other than those we showed here, and further detailed characterization of these drugs is required.

## 5 Conclusion

In this study, we biochemically characterized three agalsidases, focusing on their incorporation into cultured fibroblasts and organs/tissues of Fabry mice, and revealed the differences between them. This information will be useful not only for developing new ERT drugs but also for making a suitable ERT plan for individual Fabry patients with various backgrounds. For example, agalsidase alfa may be effective for Fabry patients in whom the disease is not so progressed and the possibility of antidrug antibody formation is low. On the other hand, agalsidase beta and agalsidase beta BS are expected to be effective for Fabry patients who have neutralizing antibodies inhibiting cellular uptake of the enzyme, which can be saturated by ERT, based on the information obtained from the previous reports [[40], [41], [42], [43]] and this study.

## Funding

Hitoshi Sakuraba has received grants from Sumitomo Pharma Co., Ltd. The funder played no role in the study design, data collection and analysis, decision to publish, or preparation of the manuscript.

## CRediT authorship contribution statement

**Takahiro Tsukimura:** Writing – original draft, Investigation. **Tomoko Shiga:** Investigation. **Tadayasu Togawa:** Writing – review & editing. **Hitoshi Sakuraba:** Writing – review & editing, Writing – original draft, Conceptualization.

## Declaration of competing interest

The authors declare that they have no known competing financial interests or personal relationships that could have appeared to influence the work reported in this paper.

## Data Availability

The authors do not have permission to share data.

## Appendix A Supplementary data

Supplementary material Image 1
