# Supplementary material for: Comparative study on incorporation of three recombinant human α-galactosidase A drugs (agalsidases) into cultured fibroblasts and organs/tissues of Fabry mice
Source: Mol Genet Metab Rep. 2024 Jul 15;40:101118. doi: 10.1016/j.ymgmr.2024.101118 (PMC11384131; doi:10.1016/j.ymgmr.2024.101118)
Supplement: Supplementary file 1 — Supplementary material [file mmc1.docx]

Supplementary Table 1. Contents of mannose 6-phosphate residues in sugar chains of agalsidases.

|  | Lee, et al.^1)^ | Sakuraba, et al.^2)^ | Morimoto, et al.^3)^ |
| --- | --- | --- | --- |
| Agalsidase alfa | 1.8 ± 0.0 (ND) | 1.3 ± 0.1 (n = 3) | NE |
| Agalsidase beta | 3.1 ± 0.1 (ND) | 3.6 ± 0.3 (n = 6) | 3.2 ± 0.2 (n = 4)* |
| Agalsidase beta BS | NE | NE | 4.2 ± 0.2 (n = 3)* |

The contents of mannose 6-phosphate residues in sugar chains are expressed as means ± standard deviation (n = number of trials).

Unit: mol/mol enzyme.

ND: not described; NE: not examined.

The monosaccharide analyses were performed by means of high-pH anion exchange chromatography, capillary electrophoresis, and anion exchange column chromatography, according to ^1)^[18], ^2)^[19], and ^3)^[20], respectively.

*These data are exhibited as a bar graph in the original report [20].
